# Encoding specificity instead of online integration of real-world spatial regularities for objects in working memory

**DOI:** 10.1167/jov.22.9.8

**Published:** 2022-08-30

**Authors:** Xinyang Liu, Ruyi Liu, Lijing Guo, Piia Astikainen, Chaoxiong Ye

**Affiliations:** 1 Institute of Brain and Psychological Sciences, Sichuan Normal University, Chengdu, China; 2 Department of Psychology, University of Jyvaskyla, Jyväskylä, Finland; 3 Faculty of Social Sciences, Tampere University, Tampere, Finland; 4 Center for Machine Vision and Signal Analysis, University of Oulu, Oulu, Finland

**Keywords:** visual working memory (VWM), spatial regularity, maintenance process, real-world object

## Abstract

Most objects show high degrees of spatial regularity (e.g. beach umbrellas appear above, not under, beach chairs). The spatial regularities of real-world objects benefit visual working memory (VWM), but the mechanisms behind this spatial regularity effect remain unclear. The “encoding specificity” hypothesis suggests that spatial regularity will enhance the visual encoding process but will not facilitate the integration of information online during VWM maintenance. The “perception-alike” hypothesis suggests that spatial regularity will function in both visual encoding and online integration during VWM maintenance. We investigated whether VWM integrates sequentially presented real-world objects by focusing on the existence of the spatial regularity effect. Throughout five experiments, we manipulated the presentation (simultaneous vs. sequential) and regularity (with vs. without regularity) of memory arrays among pairs of real-world objects. The spatial regularity of memory objects presented simultaneously, but not sequentially, improved VWM performance. We also examined whether memory load, verbal suppression and masking, and memory array duration hindered the spatial regularity effect in sequential presentation. We found a stable absence of the spatial regularity effect, suggesting that the participants were unable to integrate real-world objects based on spatial regularities online. Our results support the encoding specificity hypothesis, wherein the spatial regularity of real-world objects can enhance the efficiency of VWM encoding, but VWM cannot exploit spatial regularity to help organize sampled sequential information into meaningful integrations.

## Introduction

The capacity-limited system known as visual working memory (VWM) is vital, as it prevents the brain from being overwhelmed by information from the environment and ensures the efficient processing of incoming information (Alvarez & Cavanagh, 2004). VWM is also the foundation for higher-level cognition (Wilken & Ma, 2004). Due to its practical and theoretical importance, VWM has attracted significant interest from researchers in terms of its capacity (Fukuda, Awh, & Vogel, 2010; Luck & Vogel, 2013), function (Mičič, Horvat, & Bakracevic, 2020), and mechanisms (Heuer, Schubö, & Crawford, 2016; Mance, Becker, & Liu, 2012; Shen, Chen, Yang, Dong, Chen, & Zhou, 2021; Shen, Huang, & Gao, 2015; Shen, Tang, Wu, Shui, & Gao, 2013).

VWM studies have typically relied on memory materials made of objects or features (e.g. colors, orientations, and shapes) found in the visual environment (Gao, Li, Liang, Chen, Yin, & Shen, 2009; Luria, Sessa, Gotler, Jolicoeur, & Dell'Acqua, 2010; Woodman & Vogel, 2008). However, the way objects or features are organized, such as by color–color conjunction (Luria & Vogel, 2014) or color-orientation conjunction (Balaban & Luria, 2016), can greatly influence VWM. Many previous studies have found that participants encode targets more efficiently in their VWM when using integration principles (Hitch, Allen, & Baddeley, 2020; Li, Qian, & Liang, 2018; Peterson & Berryhill, 2013; Peterson, Gözenman, Arciniega, & Berryhill, 2015; Treisman & Zhang, 2006; Vogel, Woodman, & Luck, 2001). Among the various integration principles, Gestalt principles have been extensively examined (Wagemans, Elder, Kubovy, Palmer, Peterson, Singh, & von der Heydt, 2012a; Wagemans, Feldman, Gepshtein, Kimchi, Pomerantz, van der Helm, & van Leeuwen, 2012b).

Generally, the term “Gestalt” refers to the configuration guiding the entire integration to be immediately accessible prior to its constituent parts (von Ehrenfels, 1988). A variety of Gestalt principles, such as similarity, collinearity, and surface uniformity, have been proposed (Wertheimer, 1923), and many studies have shown that Gestalt principles elevate visual perception (Bertamini, Rampone, Makin, & Jessop, 2019; Fox, Harel, & Bennett, 2017; Kaiser, Stein, & Peelen, 2014; Stein, Kaiser, & Peelen, 2015) and VWM processes (Allon, Vixman, & Luria, 2019; Jackson & Raymond, 2008; Kałamała, Sadowska, Ordziniak, & Chuderski, 2017; Kim & Kim, 2011; Woodman, Vecera, & Luck, 2003). For example, Lin and Luck (2009) varied the similarity (homogeneity vs. heterogeneity) of colored squares to investigate the effect of the Gestalt principle. In the homogeneous condition, all colored squares belonged to the same category; consequently, those squares shared high similarity (e.g. three red squares of different shades). Conversely, in the heterogeneous condition, when the colored squares were selected from different color categories (e.g. red, blue, and green), this caused a lower level of similarity. As the results revealed, the participants showed better VWM performance in the homogeneous condition than in the heterogeneous condition. This suggests that VWM benefits from the Gestalt principle of similarity.

However, these previous studies used only single-feature objects (e.g. color; see Peterson & Berryhill, 2013) or multi-feature objects (e.g. a colored square and a tilted bar; see Balaban & Luria, 2016; Vogel et al., 2001) as memory materials, and the integration principles for the simple objects were often constrained at the perceptual level, as experience and background knowledge were not necessarily demanded or required (Kimchi, Yeshurun, & Cohen-Savransky, 2007). In contrast, the integration principles for real-world objects often exploit pre-existing information stored in long-term memory; therefore, they differ from those used for simple objects (Kaiser & Peelen, 2018; Kaiser, Quek, Cichy, & Peelen, 2019). Real-world objects are often viewed in relation to each other (e.g. spatially related); therefore, after frequent exposure to regularly organized real-world object pairs, people learn and store many so-called statistical regularities in their long-term memory (Quek & Peelen, 2020). Spatial regularity is a special type of statistical regularity (Kaiser et al., 2019) that refers to the location of one object in relation to another object (e.g. a hat on a hook). People learn the spatial contingencies of various objects through long-term exposure in daily life, so they automatically retain the objects as larger coherent unitary representations in long-term memory (Gronau, Neta, & Bar, 2008; see anti-holistic integration evidence in Markov, Utochkin, & Brady, 2021). The spatial contingencies then work as spatial regularities to reduce the memory load imposed by related real-world objects (Brady & Tenenbaum, 2013).

The possibility that objects organized according to certain integration principles may be represented beyond a linear combination of single objects in the brain has been extensively investigated (Baeck, Wagemans, & Op de Beeck, 2013; Hollingworth, 2007; Kaiser & Peelen, 2018; Kremláček, Kreegipuu, Tales, Astikainen, Põldver, Näätänen, & Stefanics et al., 2016; Quek & Peelen, 2020). By applying multi-voxel pattern analysis (MVPA) to functional magnetic resonance imaging (fMRI) data, which focuses on nonlinearity with synthetic response patterns (Kubilius, Baeck, Wagemans, & Op de Beeck, 2015), researchers are able to examine higher-level visual processing (i.e. object integration) beyond the activation of separate objects at the cortical level. Baeck et al. (2013) controlled the spatial regularity of real-world object pairs and asked participants to judge whether the object pairs were correctly positioned and whether they were likely to represent a specific action (e.g. a hammer knocking on a nail). During the task, fMRI was used to record each participant's brain activity. They found greater activity in the lateral occipital cortex (LOC) for objects with spatial regularity than for objects without regularity. More importantly, they observed a greater response to object pairs than to their constituent objects, revealing that object pairs are not represented solely as the sum of all separate objects. Kaiser and Peelen (2018) found a similar activity pattern that supported the integrative processing of object pairs in the object-selective visual cortex (OSV). These results demonstrate that the human brain is capable of exploiting integration principles to generate holistic configurations that transcend the linear processing of constituent parts.

Electrophysiological studies using electroencephalograms (EEG) and event-related potentials (ERPs) have also confirmed the effects of spatial regularity on visual perception (Quek & Peelen, 2020; Wickenden, 2014). Quek and Peelen (2020) found a greater induction of visual mismatch negativity (vMMN), an ERP component sensitive to infrequent and unexpected stimuli (Kremláček et al., 2016), for objects with spatial regularity than without regularity. These previous findings indicate that the brain is tuned to the spatial regularity of real-world objects.

Given the close relationship between perception and VWM, we can assume that for real-world objects, both perception and VWM can benefit from spatial regularity. Kaiser, Stein, and Peelen (2015) were the first to investigate the effects of the spatial regularity of objects on VWM. They used a change detection task (Luck & Vogel, 1997) to present two pairs of real-world objects with regularity (e.g. a lamp above the table, which is a typical real-life setting) or without regularity (e.g. a lamp under the table, which is an atypical real-life setting), and the participants were asked to remember the object pairs in the memory array and to report whether a change had occurred in the probe array. Better performance was found in the with-regularity condition than in the without-regularity condition. The benefit gained from spatial regularity on VWM performance is termed the “spatial regularity effect.” Importantly, the spatial regularity effect is stable, irrespective of encoding time (Kaiser, Stein, & Peelen, 2015). This suggests that spatial regularity can improve the efficiency of the VWM process for real-world objects.

Given the stability of the spatial regularity effect, the causes underlying this effect can be explained by two different hypotheses. The “perception-alike” hypothesis (Gao, Gao, Li, Sun, & Shen, 2011) holds that VWM representations, tightly intertwined with visual perceptions, can take advantage of integration principles in a similar fashion to those used for visual perception representations. Thus, even if visual stimuli disappear, the representations in VWM maintenance can still benefit from spatial regularity. Alternatively, the spatial regularity effect can also be explained by the “encoding specificity” hypothesis (Prieto, Peinado, & Mayas, 2022; Tulving & Thomson, 1973; Woodman et al., 2003), which suggests that VWM representations are determined by how information is encoded when visual stimuli appear, as visible integration cues (e.g. spatial regularity) will promote an efficient encoding of regularly organized objects into a coherent whole, whereas objects that are not regularly organized will have discrete representations. Therefore, the impact of spatial regularity on VWM processing occurs specifically in the visual encoding phase but disappears in the VWM maintenance phase. Both hypotheses provide plausible explanations for the spatial regularity effect, but they differ in whether spatial regularity plays a role in the VWM maintenance process. Kaiser et al. (2015) did not examine the specific VWM phases as influenced by spatial regularity, and, to our knowledge, no prior studies have tested the two hypotheses regarding the VWM processing of real-world objects.

One way to test these two hypotheses is to apply simultaneous and sequential presentations to manipulate the accessibility of integration principles (e.g. spatial regularity) in different process phases. In simultaneous presentation trials, integration principles are continuously available upon the appearance of the memory array, meaning that participants might integrate stimuli in the visual encoding phase. However, whether integration principles could improve the efficiency of the post-encoding phases (e.g. VWM maintenance) cannot be tested or excluded (Kałamała et al., 2017). In contrast, when stimuli are presented sequentially, participants cannot perceive the whole picture, nor can they exploit integration principles in the first display. Only in the second display, during the post-encoding phase for representations formed in the first display, can they obtain all the information and possibly integrate retained representations in the VWM with the new input.

Studies to determine which integration principles enhance VWM, such as the work by Gao, Gao, Tang, Shui, and Shen (2016), have exploited the sequential presentation condition to allow the direct investigation of the online integration of simple stimuli (a notched disk). Gao et al. (2016) compared VWM performance under with-regularity and without-regularity conditions. Two memory arrays, each containing two disks, were displayed sequentially. In the with-regularity condition, the two disks in the first array were connected to the disks in the second array via virtual elongated occluding rectangles, thereby leading to collinearity—a Gestalt principle. In contrast, in the without-regularity condition, all four disks were randomly placed. The participants showed better VWM performance (i.e. higher sensitivity) in the with-regularity condition than in the without-regularity condition. For the sequential presentation condition, the participants could only form an intact representation after all stimuli had been presented, which meant that information in separate memory arrays was integrated during the maintenance phase. Gao et al.'s (2016) work supported the beneficial effect of the Gestalt principle on the VWM maintenance phase for memory materials of simple shapes. That is, the Gestalt principle's effects can be explained by the perception-alike hypothesis rather than the encoding specificity hypothesis. However, Gao et al.'s (2016) findings cannot be directly generalized to the spatial regularity effect for real-world objects. Therefore, using the sequential presentation condition is meaningful in testing the two hypotheses regarding the spatial regularity effect.

In experiment 1 conducted for the current study, we utilized both simultaneous and sequential presentation and manipulated the regularity (with regularity vs. without regularity) to examine the spatial regularity effect on visual encoding and VWM maintenance to test the perception-alike and encoding specificity hypotheses. We predicted that the spatial regularity effect would occur in simultaneous presentation trials, which is in agreement with previous studies (Kaiser et al., 2015). That is, in the simultaneous presentation trials, VWM performance would be better under the with-regularity condition than under the without-regularity condition. The encoding specificity hypothesis suggests that the spatial regularity effect will appear only when all objects are presented simultaneously and that it will disappear when they are presented sequentially. Thus, we would observe no significant difference in VWM performance for sequentially presented object pairs between the with-regularity and without-regularity conditions. On the contrary, the perception-alike hypothesis suggests that both the encoding and maintenance phases will benefit from spatial regularity. Thus, we would expect to observe significantly better VWM performance under the with-regularity condition than under the without-regularity condition.

## Experiment 1: Examining the spatial regularity of real-world objects in VWM

In experiment 1, we tested the two hypotheses by investigating the process(es) of VWM as influenced by the spatial regularity of real-world objects. We used a change detection task and displayed real-world object pairs with or without spatial regularity, either simultaneously or sequentially. As mentioned above, in sequential presentation trials, spatial regularity exists after all the information is displayed when the encoding of objects in the first memory array has been finished and their VWM representations are available. Thus, the possible spatial regularity effect should be attributed to online integration. We also designed a simultaneous presentation condition to confirm the spatial regularity of the memory materials. We chose to use three pairs in both simultaneous and sequential presentation trials because we found a greater difference in change detection sensitivity between the with-regularity and without-regularity conditions in a previous study when the memory load was relatively high (e.g. three pairs; see Kaiser et al., 2015).

### Methods

#### Participants

Based on the previous results of the study by Gao et al. (2016), we predicted the same effect size (*η^2^_p_* = 0.34) for our experimental design. On that basis, a power analysis (G*power 3.1; Faul, Erdfelder, Lang, & Buchner, 2007) with an α level of 0.05 indicated that 14 participants were needed in our experiments to achieve 95% power. We ensured sufficient statistical power in our analyses by further increasing our sample size to 23 participants to give a sample size comparable to or larger than that used in previous similar studies (e.g. 16–23 participants for each experiment in the study by Gao et al., 2016 and 20 participants for each experiment in the study by Chen, Kocsis, Liesefeld, Müller, & Conci, 2021).

We enrolled 32 healthy undergraduates in experiment 1. Nine were excluded for not participating seriously in the experiment, as they created a very low accuracy (less than 50%, which equals the chance level). This left 23 participants (19 female subjects, 19.48 ± 0.67 years old) for further analysis. All remaining participants had normal or corrected-to-normal vision and were compensated for their participation. The experimental procedures and design complied with the Declaration of Helsinki (2008) and were approved by the ethical committee of Sichuan Normal University.

#### Stimuli and procedure

Before the formal experiments, we screened out appropriate stimuli for all experiments from prepared real-world objects by conducting a judgment task to select suitable visual materials for observing the spatial regularity effect (more details can be found in the Supplementary Materials). Similar to the objects used in the study by Kaiser et al. (2015), a set of 12 categories of real-world objects (tap, beer glass; film, tripod; pot, pot rack; mirror, sink; toilet tank, toilet; closet, clothes; bread, plate; lamp, table; range hood, gas stove; teapot, tea stove; router, set-top-box; and beach umbrella, beach chair) with spatial regularity in the vertical direction were used as stimuli. We controlled spatial regularity by organizing all object pairs in the with-regularity condition or in the without-regularity condition. We provided two different exemplars for each object, resulting in four different pairs for each category. All images were decolorized and matched for size using Photoshop 2020. Images were presented against a gray (140, 140, and 140, red, green, blue [RGB]) background on a 21-inch LCD monitor (refresh rate = 75 Hz). A single object subtended a visual angle of about 3 degrees. The experiment was programmed using E-prime 2.0.

**Figure 1.**
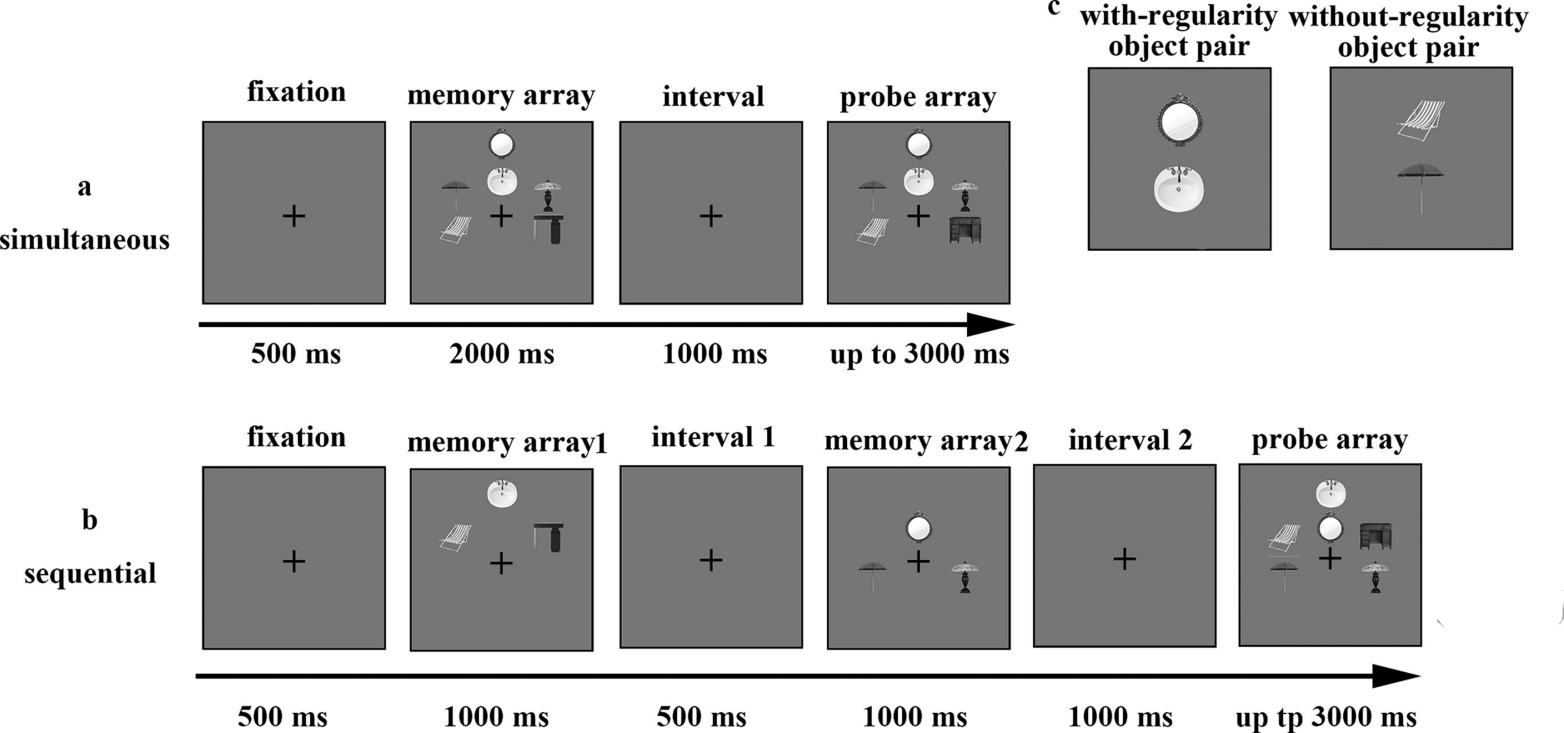
Trial structure for (**a**) simultaneous and (**b**) sequential trials and (**c**) samples of objects with/without spatial regularity in experiment 1.

The stimuli were displayed in two presentation manners: one for the simultaneous presentation condition and the other for the sequential presentation condition (Figure 1). In the simultaneous condition, a fixation cross was shown for 500 ms at the beginning of each trial. After the disappearance of the fixation cross, the memory array containing three pairs of objects (to the left, to the right, and either above or below the fixation cross) was presented for 2000 ms. After an interval of 1000 ms, the probe array, with all locations unchanged, was shown for up to 3000 ms. In 50% of the trials, the probe array was the same as the memory array, whereas in the remaining trials, one object was changed to another object within the same category. The sequential trials began with the same fixation cross, but the object pairs were presented in sequence. In the first display, one object from each of the three pairs was presented for 1000 ms, followed by a 500 ms blank screen. The second display with the other half of the pairs was shown for 1000 ms before another 1000 ms blank screen. The subsequent probe array was the same as in the simultaneous trials and contained three pairs of objects at the original locations. The participants were asked to remember the three object pairs when they all appeared simultaneously or when half of each pair appeared sequentially in the memory array. When the probe array appeared, they were required to indicate a change by pressing “K” or no change by pressing “S.” During the change detection task, accuracy was stressed rather than response speed. Each pair configuration was presented for 48 simultaneous trials and 48 sequential trials, yielding 192 trials. The experimental factors of presentation manner (simultaneous vs. sequential) and pair configuration (with-regularity object pairs vs. without-regularity object pairs) were randomly mixed within the blocks. The entire duration of experiment 1 lasted approximately 25 minutes.

#### Data analysis

The main purpose was to examine the spatial regularity effect in different presentation trials (simultaneous or sequential presentations). The sensitivity of VWM performance was calculated as the difference between hit rates and false alarm rates (i.e. a *d-*prime score [*d'* = *Z*{hit rate} – *Z*{false alarm}] was applied as an index of sensitivity toward change detection, as in Kaiser et al., 2015). A 2 (presentation manner: simultaneous vs. sequential) × 2 (pair configuration: with-regularity object pairs vs. without-regularity object pairs) repeated measures analysis of variance (ANOVA) was applied to the *d-*prime scores. Paired samples *t*-tests were conducted for the follow-up pairwise comparison between with-regularity and without-regularity object pairs within the simultaneous and sequential presentation trials. Cohen's *d* was used to estimate the effect size for the *t*-tests. The false discovery rate (FDR) corrections for *p* values of paired samples *t*-tests were calculated to control for false predictions in multiple comparisons (Benjamini & Yekutieli, 2001). JASP (version 0.16, JASP Team, 2021) was used to provide Bayes factors to show whether the *t*-test results supported the alternative hypothesis or null hypothesis (Rouder, Speckman, Sun, Morey, & Iverson, 2009; Schmalz, Manresa, & Zhang, 2021), thereby providing an odds ratio for the alternative/null hypotheses (values <1 favor the null hypothesis and values >1 favor the alternative hypothesis). The default priors in JASP were used.

### Results

#### Sensitivity (d')

**Figure 2.**
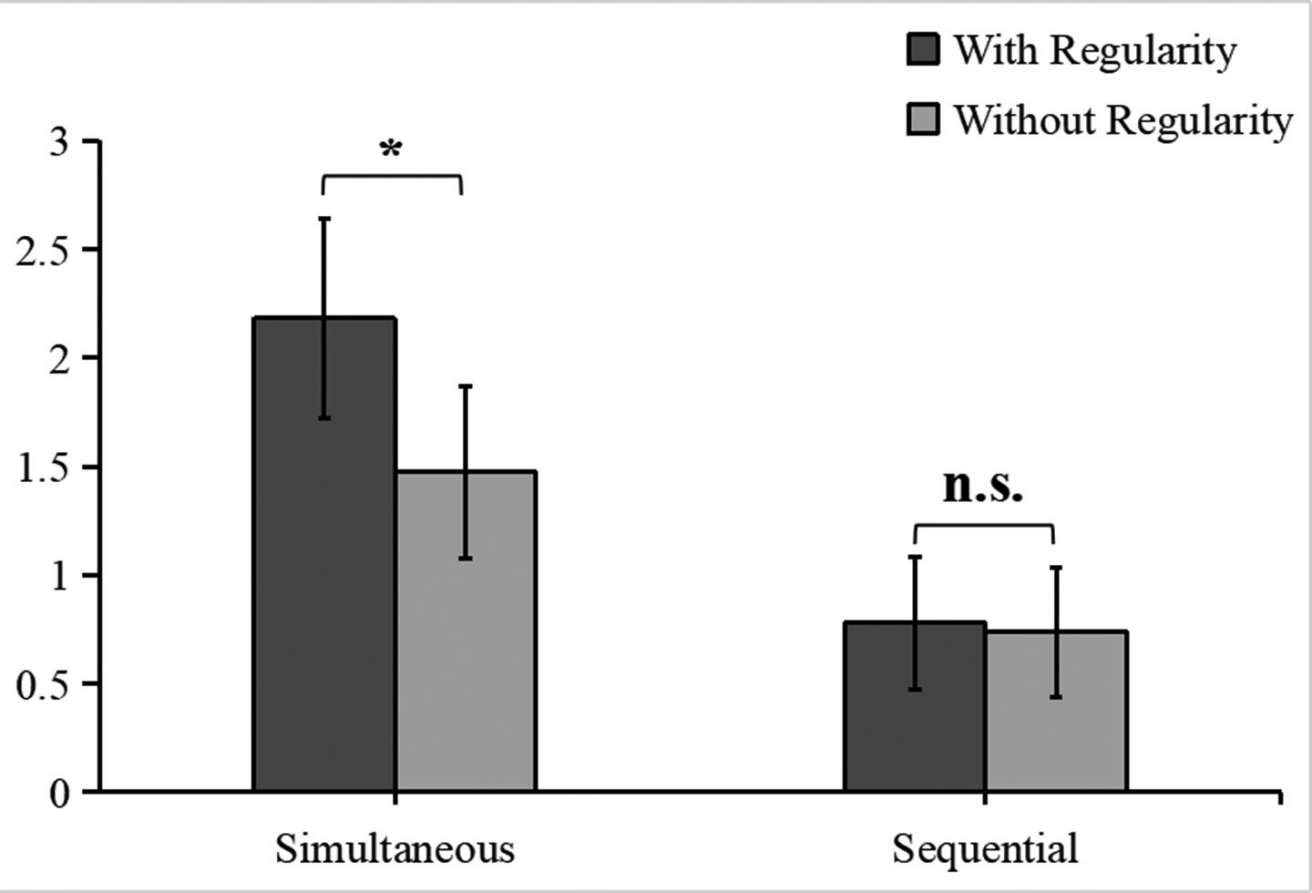
Results of experiment 1. The dark gray bars represent the with-regularity condition, and the light gray bars represent the without-regularity condition. * = *p* < 0.050, n.s. = non-significant. Error bars reflect 95% confidence intervals (CIs) (Cousineau, 2005).

The results of the 2-way repeated measures ANOVA (Figure 2) revealed a significant main effect of presentation manner, *F*(1, 22) = 43.50, *p* < 0.001, *η_p_^2^* = 0.66, and a significant interaction between presentation manner and pair configuration, *F*(1, 22) = 5.90, *p* = 0.024, *η_p_^2^* = 0.21, but no significant main effect of pair configuration, *F*(1, 22) = 3.45, *p* = 0.077, *η_p_^2^* = 0.14. Planned comparisons revealed that in the simultaneous presentation trials, the sensitivity was significantly higher in the with-regularity condition than in the without-regularity condition, *t*(22) = 2.52, *p* = 0.038, Cohen's *d* = 0.67, BF_10_ = 2.82. In contrast, in the sequential presentation trials, no significant difference was detected between the with-regularity and without-regularity conditions, *t*(22) = 0.21, *p* = 0.838, Cohen's *d* = 0.06, BF_10_ = 0.22.

### Discussion

In experiment 1, we found that the participants had a higher sensitivity to with-regularity than to without-regularity object pairs in the simultaneous presentation trials. These results indicate that when stimuli were presented simultaneously, spatial regularity enhanced the participant's VWM performance, thereby confirming the stability of the spatial regularity effect found in the study by Kaiser et al. (2015). This also suggests that the object pairs used in our study were effective, as the spatial regularity effect occurred in the simultaneous presentation trials. One possible reason for the effect observed in the simultaneous presentation condition is that the configuration of regularly organized object pairs is readily accessible; therefore, it can forge a stronger sensory impression than object pairs without regularity. A stronger sensory impression improves the encoding of the object pairs, thereby leading to better VWM performance.

However, in the sequential presentation trials, sensitivity was not significantly different between the with-regularity and without-regularity object pairs. No spatial regularity effect occurred when the real-world objects appeared sequentially, suggesting that spatial regularity failed to enhance the online integration of VWM representations. In general, the results seem to favor the encoding specificity hypothesis.

Furthermore, these results seem to contradict Gao et al.'s (2016) findings that participants had a higher sensitivity to with-regularity than to without-regularity object pairs in sequential presentation trials. A main cause of this contradiction may be the different stimuli used in the two studies. Previous research has demonstrated that an individual's VWM storage capacity is reduced with an increase in stimulus complexity (Alvarez & Cavanagh, 2004), such as two faces (Eng, Chen, & Jiang, 2005) or three to four colors (Luck & Vogel, 1997). Gao et al. (2016) used simple disks as memory materials, and their participants remembered all the disks in the two sequential displays. However, in the current study, participants may have formed VWM representations of two complex real-world objects in each of the two memory arrays while retaining the remaining objects as conceptual representations in their minds. Spatial regularity specifically influences visual processes; therefore, when the memory load exceeds the VWM capacity, participants may integrate only one pair of objects displayed in the two arrays due to occasionally discrepant types of representations (e.g. encoding a teapot and a closet in the first display but the set-top box and clothes in the second one, thereby incurring integration failure for two objects; Bays, 2016).

Representation discrepancy caused by the exceeding of the memory load might weaken the impact of spatial regularity on VWM performance in sequential presentation trials. Previous studies have indicated that the perceptual integration principles for simple objects are stimulus driven and capture attention automatically (Kimchi et al., 2007). The collinearity effect examined by Gao et al. (2016) was a low-level perceptual integration principle that demanded fewer cognitive resources (i.e. subjective attention) than the complex integration principles for real-world objects, which rely on an individual's attentional capacity (Gronau & Shachar, 2014) and depend on experience and long-term memory (Kaiser et al., 2019). In addition, only two disks were displayed by Gao et al. (2016) in each memory array, and this was within each individual's VWM capacity. That is, the memory load was lower in Gao et al.'s (2016) study than in experiment 1 of the present study. Therefore, the overburdening of memory from real-world objects might make the use of spatial regularity more difficult for participants during sequential presentation trials because of the integration failure this causes between the different types of representations.

In experiment 2, we tested whether the memory load might influence the occurrence of the spatial regularity effect by specifically controlling the memory load and using only sequential presentations. If the integration of real-world objects presented sequentially is too difficult, no significant difference should occur between the with-regularity and without-regularity conditions, whether with high or low memory loads. On the contrary, if the overburdened memory is what weakens the spatial regularity effect, a reduction in the memory load should lead to the occurrence of the spatial regularity effect in sequential presentation trials.

## Experiment 2: Examining the effect of memory load on the spatial regularity of real-world objects in VWM

In experiment 1, we used three pairs of real-world objects as the memory materials in each trial and found no significant difference between with-regularity objects and without-regularity objects in the sequential presentation trials, supporting the encoding specificity hypothesis that only the encoding phase benefits from spatial regularity. However, the memory load might exceed each individual's VWM capacity; therefore, the sequential presentation of the stimuli might cause integration failure for VWM representations with conceptual representations in the two displays, thereby hindering the spatial regularity effect. That is, we cannot accept the encoding specificity hypothesis unless we were to also exclude the possibility that the overburdened memory would lead to the absence of the spatial regularity effect in sequential presentation trials.

In experiment 2, we exploited the sequential presentation condition in experiment 1 and manipulated the memory load of the stimuli. Because the spatial regularity effect had been verified in experiment 1, the use of a sequential stimulus presentation was sufficient to test whether spatial regularity facilitated online integration. In addition to the three object-pair trials, we added a low memory load condition of two object pairs to explore the potential impact of memory load on the spatial regularity effect.

### Experiment 2a

#### Methods

##### Participants

As a follow-up experiment to experiment 1, we set a comparable sample size in experiment 2a. We recruited a new group of 26 undergraduates. Two participants were excluded from further analysis because they did not participate seriously in the experiment, leading to very low accuracy (less than 50%, which equals the chance level). This left a final group of 24 participants (22 female subjects, 19.38 ± 1.50 years old). All participants had normal or corrected-to-normal vision and were compensated for their participation. The experimental procedures and design complied with the Declaration of Helsinki (2008) and were approved by the ethical committee of Sichuan Normal University.

##### Stimuli and procedure

**Figure 3.**
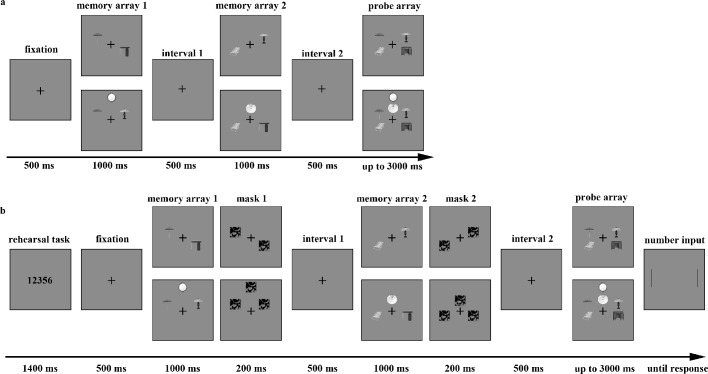
The structure of a change trial in (**a**) experiment 2a and (**b**) experiment 2b.

The stimuli and apparatuses in experiment 2a were identical to those in experiment 1. Because previous studies suggest that VWM performance improves when the objects to be remembered are distributed between the left and right visual fields compared to when they are all presented within the same hemifield (Delvenne, 2005; Umemoto, Drew, Ester, & Awh, 2010; Zhang, Ye, Roberson, Zhao, Xue, & Liu, 2018), we ensured that all items were presented bilaterally in each memory array. The main procedure in experiment 2a (Figure 3) was the same as in experiment 1, except that only the sequential presentation trials were applied and the factor of memory load was manipulated. We set two memory loads: the lower level involved two pairs, and the higher level involved three pairs. The stimuli locations on screen in the higher-level trials were the same as in the sequential presentation trials of experiment 1, whereas in the lower-level trials, the objects were presented to the left and right of the fixation cross, and the two objects in the same display were arranged diagonally. As in experiment 1, experiment 2a consisted of 192 trials, with each pair configuration presented for 48 two-pair trials and 48 three-pair trials (24 change trials and 24 without-change trials for each condition). The experimental factors of memory load (two vs. three object pairs) and pair configuration (with-regularity vs. without-regularity object pairs) were randomly mixed within the blocks. The entire duration of experiment 2a lasted approximately 30 minutes.

##### Data analysis

As in experiment 1, we calculated the *d-*prime scores as an index of sensitivity toward change detection. We also applied a 2 (memory load: two-pair vs. three-pair) × 2 (pair configuration: with-regularity object pairs vs. without-regularity object pairs) repeated measures ANOVA to the *d-*prime scores. The follow-up pairwise comparison of different pair configurations under the two memory load conditions was conducted using paired samples *t*-tests. For both the *d-*prime scores, Cohen's *d* was used to estimate the effect size for the *t*-tests. The Bayes factors for the *t*-tests are reported.

#### Results

##### Sensitivity (d′)

**Figure 4.**
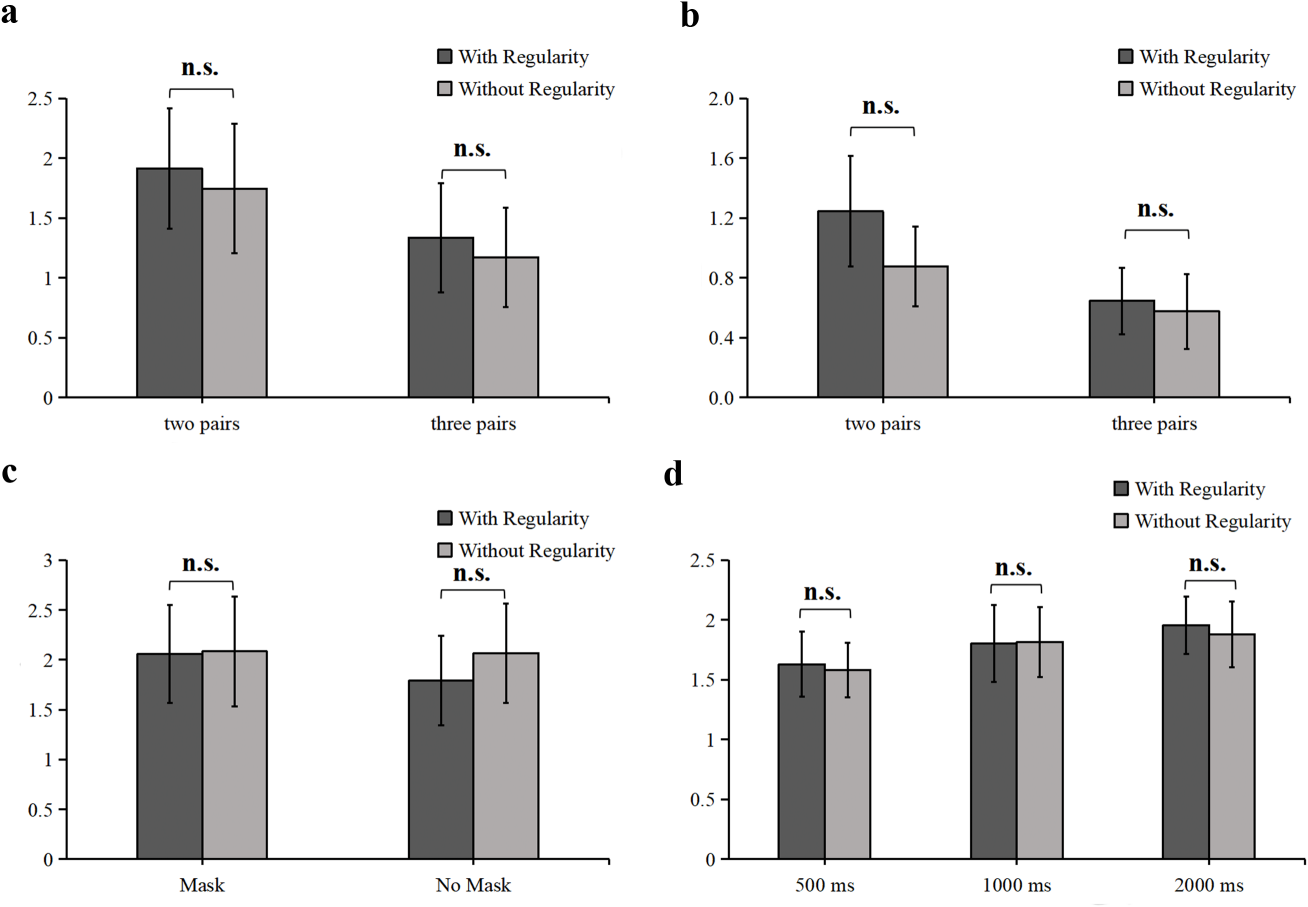
Results of (**a**) experiment 2a, (**b**) experiment 2b, (**c**) experiment 3, and (**d**) experiment 4. The dark gray bars represent the with-regularity condition, and the light gray bars represent the without-regularity condition. n.s. = non-significant. Error bars reflect 95% confidence intervals (CIs).

A 2-way repeated measures ANOVA of the *d*-prime scores (Figure 4) revealed no significant interaction between memory load and pair configuration, *F*(1, 23) < 0.01, *p* = 0.985, *η_p_^2^* < 0.01, and no significant main effect of pair configuration, *F*(1, 23) = 0.38, *p* = 0.542, *η_p_^2^* = 0.02. A significant main effect of memory load was found, *F* (1, 23) = 8.10, *p* = 0.009, *η^2^* = 0.26, with a greater sensitivity for the two-pair condition than for the three-pair condition, *t*(23) = 2.85, *p* = 0.009, Cohen's *d* = 0.58, BF_10_ = 5.21. The results of the paired samples *t*-tests revealed no significant difference between with-regularity and without-regularity object pairs in the two-pair condition, *t*(23) = 0.47, *p* = 0.646, Cohen's *d* = 0.13, BF_10_ = 0.24, or in the three-pair condition, *t*(23) = 0.67, *p* = 0.513, Cohen's *d* = 0.15, BF_10_ = 0.26.

#### Discussion

In experiment 2a, the sensitivity did not differ independently between the with-regularity and without-regularity object pairs, regardless of whether the memory load was high or low. These results seem to exclude the impact of an overburdened memory load, as the two-pair condition yielded no spatial regularity effect. In line with our findings in experiment 1, experiment 2a also aligned with previous studies that found worse memory performance for spatial layouts in which the objects were presented sequentially rather than simultaneously (Liu, 2010). This result can be explained by the difficulty associated with integrating objects online.

The results of experiment 2a revealed no spatial regularity effect during the maintenance process; however, this does not mean that online integration is impossible. The 1000 ms duration for displaying the memory array is relatively long compared to the time settings used in previous experiments, which applied simple features (Balaban & Luria, 2016; Kałamała et al., 2017). Therefore, participants might have used verbal memory to help memorize the object pairs, regardless of their spatial regularity. Previous studies that used real-world objects as stimuli usually used a verbal suppression task to interrupt verbal rehearsal (Kaiser et al., 2015). Moreover, Gao et al. (2016) thought that the sensory afterimages of a stimulus in the first display might still occur after the onset of the second display if the blank screen interval between the two sequential presentations was too short (e.g. 500 ms). The blank screen interval was relatively short (500 ms) in the current study; therefore, images appearing later in the retina might serve as novel stimuli that occupy more attention. A salient local stimulus would interfere with global processing (Weinbach & Henik, 2014), which could obstruct integration. The spatial regularity effect demands more cognitive resources and individual experience than the Gestalt principal effect, so it is influenced more by sensory afterimages. Therefore, experiment 2b was performed to exclude the interference of verbal rehearsal and sensory afterimages.

### Experiment 2b

In experiment 2b, we added a verbal suppression task at the beginning of the procedure and provided masks immediately after the stimulus disappeared. Real-world object pairs were used as stimuli in the current study, and participants might have taken advantage of semantic coding to help memorize the object pairs. However, spatial regularity only benefits visual processes. Semantic coding might hinder the spatial regularity effect and could be validly inhibited by a verbal suppression task, as previous research has shown (Kaiser et al., 2015). In addition, two scrambled images were displayed as masks after each of the memory arrays, as described by Gao et al. (2016), to eliminate sensory afterimages.

#### Methods

##### Participants

We set a sample size in experiment 2b comparable to that in experiment 1. We recruited a new group of 29 undergraduates. Four participants were excluded from the analysis. Two of them did not finish the experiment; the other two had poor performance (accuracy <50%), leaving a final group of 25 participants (19 female subjects, 19.76 ± 1.54 years old) for further analysis. All participants had normal or corrected-to-normal vision and were compensated for their participation. The experimental procedures and design complied with the Declaration of Helsinki (2008) and were approved by the ethical committee of Sichuan Normal University.

##### Stimuli and procedure

The stimuli used in experiment 2b were identical to those used in experiment 2a. The main procedure in experiment 2b (see Figure 3) was the same as in experiment 2a. Importantly, to prevent the possible use of verbal memory to encode the objects, we took a similar approach to that described by Kaiser et al. (2015). Five-digit numbers were presented at the beginning of the trial for 1400 ms, and the participants were asked to rehearse the numbers throughout the trial. After the presentation of the first and second memory arrays, masks were shown for 200 ms at the same locations the stimuli were in the memory arrays to reduce the impact of iconic memory. A total of 192 trials were conducted in experiment 2b, with the experimental factors of memory load (two pairs vs. three pairs) and pair configuration (with-regularity vs. without-regularity object pairs) randomly mixed within the blocks. The entire duration of experiment 2b lasted approximately 40 minutes.

##### Data analysis

All analyses were identical to those described in experiment 2a. Because the participants had nearly perfect performance in the verbal suppression task (mean accuracy ≥98%), we kept all trials for further analysis.

#### Results

A 2-way repeated measures ANOVA of *d*-prime scores (see Figure 4) revealed no significant interaction between memory load and pair configuration, *F*(1, 24) = 1.23, *p* = 0.278, *η_p_^2^* = 0.05, and no significant main effect of pair configuration, *F*(1, 24) = 2.57, *p* = 0.122, *η_p_^2^* = 0.10. A significant main effect was observed for memory load, *F*(1, 24) = 12.46, *p* = 0.002, *η^2^* = 0.34, with higher sensitivity for the two-pair condition than for the three-pair condition, *t*(24) = 3.53, *p* = 0.002, Cohen's *d* = 0.83, BF_10_ = 21.73. No significant difference was found between the with-regularity and without-regularity pairs in the two-pair condition, *t*(24) = 1.60, *p* = 0.123, Cohen's *d* = 0.44, BF_10_ = 0.64, or in the three-pair condition, *t*(24) = 0.50, *p* = 0.620, Cohen's *d* = 0.12, BF_10_ = 0.24.

#### Discussion

The results of experiment 2b again showed no significant difference between with-regularity and without-regularity object pairs presented sequentially, regardless of the memory load, thereby indicating that verbal rehearsal and sensory afterimages had no influence on the pattern of results in experiment 2a. Previous studies using fMRI found the dissociated activation of verbal working memory and VWM in the frontal and parietal cortex (Ikeda & Osaka, 2007; Rothmayr, Baumann, Endestad, Rutschmann, Magnussen, & Greenlee, 2007), demonstrating two relatively independent subsystems for working memory.

Some previous studies suggest that individual differences in VWM capacity can explain the variance in attention resource allocation ability across individuals (Cowan & Morey, 2006; Fukuda & Vogel, 2009; Vogel, McCollough, & Machizawa, 2005), whereas a recent study found no support for the claim that individual differences in VWM capacity are related to the internal attention allocation (Ye, Xu, Liu, Astikainen, Zhu, Hu, & Liu, 2021). In addition, the VWM capacity actually fluctuates among individuals (Cowan, 2001); therefore, our arranged memory loads might have exceeded the capacities of some participants, thereby concealing the spatial regularity effect in the other part of the sample. We tested the interruption to the individual capacity difference by asking the participants in experiment 2b to complete a VWM capacity measurement after they had completed the main task. However, the results indicated that individual differences in VWM capacity had no influence on the degree of online integration, regardless of the memory load (more details can be found in the Supplementary Materials).

Taken together, the results in experiment 2 favor the idea that the overburdened memory has no impact on the disappearance of the spatial regularity effect in sequential presentation trials. However, as shown in Figure 4, the results of experiment 2b showed that when participants needed to remember two pairs of stimuli, the *d*-prime scores seemed better in the with-regularity condition than in the without-regularity condition (although no statistically significant difference was detected between them). One possible reason for this is that the spatial regularity effect may be more obvious when the memory load consists of two pairs. In addition, some relatively deficient controls might have impeded the spatial regularity effect. Therefore, we still cannot directly conclude that participants were unable to integrate objects with spatial regularity. Although we selected these materials based on a judgment task (see Supplementary Materials) and our instructions encouraged participants to pair the memory objects, the participants might have failed to pair them due to the absence of specific experiences (e.g. a participant may never have seen a gas stove in daily life) or of the desire to pair them in the formal experiment. Moreover, participants may have inadvertently tried to integrate the objects presented in the same display, and with obvious failure, they were unaware of the spatial regularity. Because the participants did not have an expectation as to which pair of stimuli to integrate, they may have integrated the stimuli incorrectly or memorized each object independently (Bays, 2016). Therefore, in the follow-up experiments, we delineated the location of each pair with a bounding box to eliminate the ambiguity of integration.

Because the results of experiments 2a and 2b seemed to show slightly different trends, in experiment 3, we tested whether verbal working memory and sensory afterimages would again impair the spatial regularity effect in sequential trials. If the participants’ performances improved under the with-regularity condition compared to the without-regularity condition after adding a verbal suppression task and masks, then those two factors influenced the online integration of real-world objects. On the contrary, if no significant difference was evident between the pair-configuration trials in either experimental design conditions, then the failure of online integration using spatial regularities is reliable, and the results would support the encoding specificity hypothesis.

## Experiment 3: Examining the effects of verbal working memory and sensory afterimages on the spatial regularity of real-world objects in VWM

In experiment 2b, controlling the memory load of real-world objects and utilizing a sequential presentation with a verbal suppression task and masks revealed only a trend of difference between the with-regularity and without-regularity conditions. In consideration of the individual difference in spatial regularity effect, we tested whether the absence of the spatial regularity effect in sequential trials was stable, despite the rigorous control of verbal working memory and sensory afterimages, by combining experiments 2a and 2b into a within-subject experiment in experiment 3. Because the trend seemed more obvious in the lower memory load condition, we fixed the stimuli in the memory arrays as two-pair objects. We eliminated the ambiguity when associating two objects in the same display by adding a box around each corresponding object pair.

We also asked the participants to engage in a judgment task before the main task to confirm their familiarity with the spatial regularities of each pair of objects. The judgment task could also have improved participants’ familiarity with the stimuli and increased their motivation to integrate objects in the main follow-up task. Very high accuracy in the judgment task (i.e. a ceiling effect) indicated that the participants were familiar with the spatial regularity and were precise about the spatial relationship discrimination.

### Methods

#### Participants

We set a sample size in experiment 3 comparable to that in experiment 1. We recruited a new group of 25 undergraduates. Two participants were excluded from further analysis due to extremely low accuracy (less than 50%), which left a final group of 23 participants (23 female subjects, 19.13 ± 1.29 years old). All participants had normal or corrected-to-normal vision and were compensated for their participation. The experimental procedures and design complied with the Declaration of Helsinki (2008) and were approved by the ethical committee of Sichuan Normal University.

#### Stimuli and procedure

The stimuli and apparatuses in experiment 3 were the same as those in experiments 1, 2a, and 2b.

We first showed the participants all the object pictures used in experiment 3, and we asked them to say the objects’ names out loud in cases of incognizance. When familiarity with the objects was ensured, the participants commenced the judgment task in which two objects were presented on the screen. We manipulated the regularity of the object pairs to present two levels of stimuli: with-regularity pairs (96 trials) and no-regularity pairs (96 trials). Participants pressed “1” if objects had spatial regularity and pressed “2” otherwise. Accuracy was prioritized over speed. The entire judgment task lasted for approximately 20 minutes.

**Figure 5.**
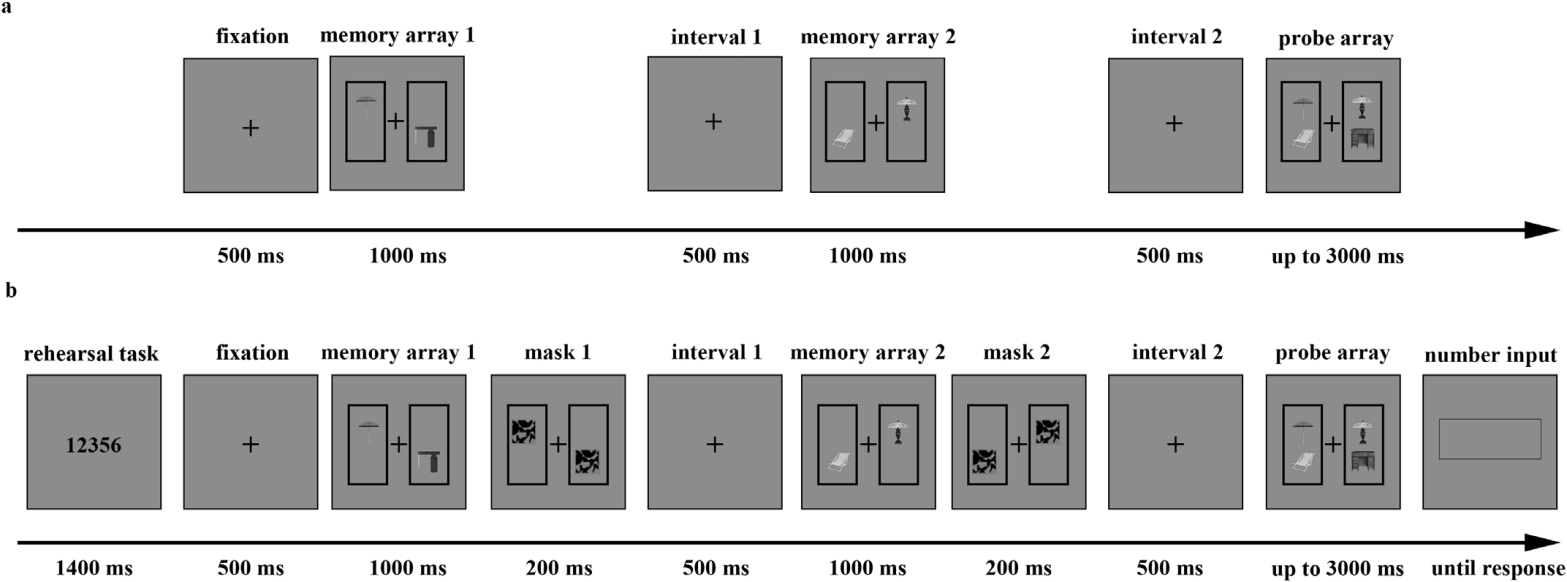
The structure of the (**a**) change trial without verbal suppression or masks and (**b**) change trial with verbal suppression and masks in experiment 3.

Upon completion of the judgment task and after a short break, the participants started the main task. In the main task (see Figure 5), two 1000 ms memory arrays were presented after a 500 ms fixation. The first memory array contained two objects sorted into different object pairs and were presented in a box to the left or right of the fixation cross (the horizontal distance between the left and right pairs of stimuli was a 6 degree visual angle). The other half of the object pairs was presented in the second memory array after a 500 ms interval. The participants were instructed that the two objects appearing sequentially in the box at the same locations could be regarded as pairs. In half of the trials, the object pairs had spatial regularity, whereas they were reversed in the other half. After a second 500 ms interval, the test array provided two object pairs, each in a box, at the same locations as the memory arrays. The participants had to decide whether the probe objects were identical to the memory objects; if they were identical, the participants were asked to press “S”; otherwise, they were to press “K.” We emphasized that only one object would change and that the change was intra-category instead of inter-category in the change trials. The probe objects in half of the trials were identical to the memory objects and changed in the other half. The test array lasted up to 3000 ms and disappeared after the participants pressed a key. Accuracy was prioritized over speed. Moreover, in half of the trials, we also presented a 1400 ms rehearsal task display at the beginning and a 200 ms mask immediately after the memory arrays.

The main task of experiment 3 was similar to the composite of experiments 2a and 2b, except for four changes. First, we adjusted the existence of verbal suppression tasks and masks into a within-subject condition between blocks. Second, we asked the participants to memorize only two pairs. Third, we added two boxes surrounding the object pairs to the left and right of the cross throughout the memory and test arrays. Fourth, before the main experiment, the participants completed a judgment task in which they were asked to assess the spatial relationships between the objects presented as pairs. The aims of this task were to improve each participant's ability to distinguish spatial regularity and to check their familiarity with the spatial regularities of each pair of objects.

The experimental design conditions (with verbal suppression and masks vs. without verbal suppression or masks) were blocked, with their order counterbalanced across subjects. Stimuli with each pair configuration (condition) were randomly presented within the blocks, yielding 96 trials for each condition. A total of 192 trials with four blocks were conducted, and the entire experiment took approximately 40 minutes.

#### Data analysis

We calculated the *d-*prime scores as an index of sensitivity toward change detection and used a 2 (experimental design: with verbal suppression and masks vs. without verbal suppression or masks) × 2 (pair configuration: with-regularity object pairs vs. without-regularity object pairs) repeated measures ANOVA to determine the *d-*prime scores. The follow-up pairwise comparison of different pair configurations under the two experimental design conditions was conducted through paired samples *t*-tests. For the *d-*prime scores, Cohen's *d* was used to estimate the effect size for the *t*-tests. The Bayes factors for the *t*-tests are reported.

#### Results

##### Accuracy of the judgment task

The accuracy of the judgment task (0.94 ± 0.04) was very high, indicating a ceiling effect.

##### Sensitivity (d′’)

The results of the 2-way repeated measures ANOVA (see Figure 4) revealed no significant interaction between experimental design and pair configuration, *F*(1, 22) = 0.52, *p* = 0.478, *η_p_^2^* = 0.02, nor a significant main effect of experimental design, *F*(1, 22) = 0.43, *p* = 0.518, *η_p_^2^* = 0.02, or pair configuration, *F*(1, 22) = 0.62, *p* = 0.440, *η_p_^2^* = 0.03. The results of paired samples *t*-tests revealed no significant difference between with-regularity and without-regularity object pairs in the trials with rehearsal tasks and masks, *t*(22) = 0.10, *p* = 0.920, Cohen's *d* = 0.02, BF_10_ = 0.22, or in the trials without rehearsal tasks or masks, *t*(22) = 1.10, *p* = 0.283, Cohen's *d* = 0.24, BF_10_ = 0.38.

#### Discussion

In experiment 3, participants were familiar with the provided spatial regularity, as indicated by the near-perfect accuracy result for the judgment task, but the sensitivity to the different pair-configuration stimuli still showed no significant differences. This indicates that there was a consistent absence of the spatial regularity effect in sequential presentations, even though we meticulously controlled for verbal working memory and sensory afterimages. The within-subject design directly revealed that rehearsal tasks and masks had no impact on the online processing of real-world objects in the VWM.

The results of experiments 1 to 3 support the encoding specificity hypothesis that participants can only use spatial regularity in the encoding phase of VWM. We utilized simultaneous and sequential presentations to test the spatial regularity effect of real-world objects and the phase of action in experiment 1. Subsequently, experiments 2a, 2b, and 3 excluded some variables that might have impeded the online use of spatial regularity. Specifically, these variables were excessive memory load, the aid of verbal working memory, sensory afterimages, the motivation for integration, and individual differences.

Apart from these potentially impeding factors in the sequential trials, the longer display durations in the simultaneous condition in experiment 1 presumably facilitated the integration of paired objects and elicited the spatial regularity effect. We matched the total encoding time by choosing 2000 ms for each memory array in the simultaneous trials (with three paired objects) and 1000 ms for each memory array in the sequential trials (with three single objects). However, previous studies have shown that participants can allocate VWM resources in different ways for different display durations (Long, Ye, Li, Tian, & Liu, 2020; Ye, Hu, Li, Ristaniemi, Liu, & Liu, 2017; Ye, Liang, Zhang, Xu, Zhu, & Liu, 2020; Ye, Sun, Xu, Liang, Zhang, & Liu, 2019). The longer display durations in the simultaneous condition could have led to more inevitable passive eye movements, which would have enhanced the goal locations and provided memory traces in the VWM (Damiano & Walther, 2019; Hanning & Deubel, 2018). Furthermore, they might have enabled the participants to take advantage of spatial regularity.

In experiment 4, we manipulated the display duration of the sequential trials to investigate whether the presentation time of the memory arrays affected the online integration of real-world objects. If the performance was significantly better with with-regularity objects than without-regularity objects in a long-display-duration condition (2000 ms for each memory array) and if the performances between the pair-configuration conditions revealed no significant differences in short-display-duration trials (500 ms for each memory array), more eye movements could be vital in causing the spatial regularity effect. If no spatial regularity effect arose despite the systematic manipulation of display duration, eye movements probably had no impact on online integration, and our results would sustain the encoding specificity hypothesis.

## Experiment 4: Examining the effects of display duration on the spatial regularity of real-world objects in VWM

Because eye movements might strengthen participants’ use of spatial regularity, we implemented three different display durations for each memory array to test whether spatial regularity appeared in sequential trials. Prior research has confirmed that 500 ms is sufficient to encode two complex stimuli (e.g. faces in Ye, Xu, Liu, Cong, Saariluoma, Ristaniemi, & Astikainen, 2018). We also used a judgment task to improve familiarity with spatial regularity and the motivation to integrate. Therefore, 500 ms per memory array was chosen as the short display duration and 2000 ms as the long duration to correspond to the display durations used in the simultaneous trials in experiment 1. In experiment 3, we demonstrated that verbal suppression and masks had no impact on the success of online integration; thus, we removed these controls in experiment 4 to simplify the task.

In addition, in experiment 3, we added boxes around the memory array to guide the participants to realize which pair of objects they needed to integrate. However, because the boxes only appeared when the memory array appeared and disappeared with the disappearance of the memory array, the sudden appearance and disappearance of the boxes could have attracted additional participant attention. Therefore, the setting of bounding boxes around memory items could have interfered with the integration of paired objects. In experiment 4, bounding boxes were kept on the screen throughout the experiment to reduce possible interference with the integration caused by the appearance and disappearance of the boxes.

### Methods

#### Participants

We set a sample size in experiment 4 comparable to that in experiment 1. We enrolled 25 healthy undergraduates. Two of them were excluded for a lack of serious participation, which created very low accuracy (no more than 50%, which equals the chance level). This left 23 participants (21 female subjects, 20.22 ± 1.83 years old) for further analysis. All remaining participants had normal or corrected-to-normal vision and were compensated for their participation. The experimental procedures and design complied with the Declaration of Helsinki (2008) and were approved by the ethical committee of Sichuan Normal University.

#### Stimuli and procedure

The stimuli and apparatuses in experiment 4 were the same as in experiments 1 to 3. As in experiment 3, we initially showed the participants all the object pictures used in experiment 4 and asked them to say the objects’ names out loud in cases of incognizance. The participants engaged in the same judgment task as in experiment 3. Upon completion of the judgment task and after a short break, the participants were asked to complete the main task.

**Figure 6.**
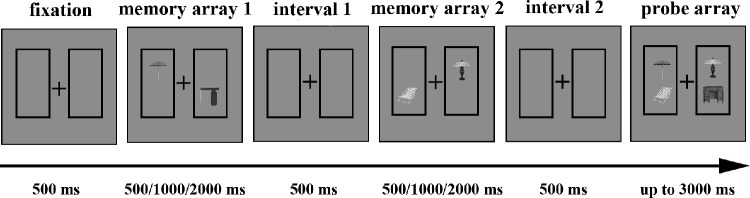
The structure for trials with regularity in experiment 4.

The main task of experiment 4 was similar to the trials without verbal suppression or masks in experiment 3 (see Figure 6), except that we changed the display duration (500 ms vs. 1000 ms vs. 2000 ms) as a variable and kept the boxes around the paired objects throughout the trial to decrease their interference. After the judgment task, the participants completed 32 trials for each condition, with a total of 192 trials randomly organized into four blocks. The entire task lasted approximately 40 minutes.

#### Data analysis

We calculated the *d-*prime scores as an index of sensitivity to change detection. A 2 (pair configuration: with-regularity vs. without-regularity) × 3 (display duration: 500 ms vs. 1000 ms vs. 2000 ms) repeated measures ANOVA was applied to the *d-*prime scores. Follow-up pairwise comparisons of different pair configurations under three display-duration conditions were conducted using paired samples *t*-tests. For the *d-*prime scores, Cohen's *d* was used to estimate the effect size for the *t*-tests. The Bayes factors for the *t*-tests are reported.

#### Results

##### Accuracy of the judgment task

The accuracy of the judgment task (0.98 ± 0.04) was very high, indicating a ceiling effect.

##### Sensitivity (d′)

A 2-way repeated measures ANOVA of the *d*-prime scores (see Figure 4) revealed no significant interaction between display duration and pair configuration, *F*(2, 44) = 0.08, *p* = 0.928, *η_p_^2^* < 0.01, and no significant main effect of pair configuration, *F*(1, 22) = 0.36, *p* = 0.552, *η_p_^2^* = 0.02. A significant main effect of display duration was found, *F* (2, 44) = 5.23, *p* = 0.009, *η_p_^2^* = 0.19, with greater sensitivity under the 2000 ms condition than under the 500 ms condition, *t*(22) = 3.81, *p* = 0.009, Cohen's *d* = 0.80, BF_10_ = 36.58. The results of paired samples *t*-tests revealed no significant differences between with-regularity and without-regularity object pairs in each display-duration condition; 500 ms duration: *t*(22) = 0.32, *p* = 0.752, Cohen's *d* = 0.08, BF_10_ = 0.23; 1000 ms duration: *t*(22) = 0.09, *p* = 0.933, Cohen's *d* = 0.02, BF_10_ = 0.22; and 2000 ms duration: *t*(22) = 0.70, *p* = 0.489, Cohen's *d* = 0.12, BF_10_ = 0.27.

##### Combined analysis of the spatial regularity effect under the sequential presentation condition

In addition to the analyses mentioned above, we quantified the spatial regularity effect by calculating the spatial regularity index (SRI) by subtracting the *d*-prime score for the without-regularity condition from the *d*-prime score for the with-regularity condition. The SRI was calculated using the following formula:

$$\mathrm{SRI}=d{}_{\mathrm{with}-\mathrm{regularity}}^{'}-d{}_{\mathrm{without}-\mathrm{regularity}}^{'}$$

In the formulation, *d*′_with − regularity_ refers to sensitivity under the with-regularity condition and *d*′_without − regularity_ refers to sensitivity under the without-regularity condition. Therefore, an SRI larger than zero suggests better performance under the with-regularity condition than under the without-regularity condition, and vice versa. An SRI equal to zero indicates that spatial regularity has no impact on VWM.

We confirmed the presence of a spatial regularity effect in the sequential presentation trials by combining the SRI values in the five formal experiments (only sequential presentation condition in experiment 1, averaged between the two-pair and three-pair conditions in experiment 2a or experiment 2b, averaged between the with and without verbal suppression and mask conditions in experiment 3, and averaged between the three display-duration conditions in experiment 4) to obtain a large sample of data (*n* = 118). A mean SRI was calculated for each participant in the sample, and an independent samples *t*-test was conducted to compare the SRI (0.005 ± 0.61) against zero. Again, the results revealed no significant difference between the mean SRI and zero, *t*(117) = 0.10, *p* = 0.924, Cohen's *d* = 0.01, BF_10_ = 0.10.

#### Discussion

In experiment 4, the participants were also familiar with the provided spatial regularity according to the near-perfect accuracy results for the judgment task. Furthermore, the significantly better performance in the trials with a 2000 ms than 500 ms display duration implied a VWM facilitation of a longer encoding time. Interestingly, the *d*-prime results for experiment 4 were consistent with those in experiments 1 to 3, indicating no significant difference between with-regularity and without-regularity object pairs in the sequential presentation condition, despite the increase in passive eye movements. These results are in accordance with other research demonstrating that eye movements do not enhance VWM during the encoding or maintenance phases (Lange & Engbert, 2013; Martin, Tapper, Gonzalez, Leclerc, & Niechwiej-Szwedo, 2017).

The SRIs of the sequential presentation trials in all experiments showed the same pattern; therefore, we calculated the mean SRI of these experiments to conduct a combined analysis based on a relatively large sample. Again, we found no significant difference between the two pair-configuration trials, and this result provided strong evidence that online integration was not likely to occur within the domain of real-world stimuli.

## General discussion

In the current study, we replicated the results of a previous study (Kaiser et al., 2015) that showed that participants had a higher sensitivity to the with-regularity than without-regularity object pairs. This result indicates that participants can exploit the spatial regularities of real-world objects to improve their VWM performance.

However, our study differs from the study by Kaiser et al. (2015) in one important aspect: Kaiser et al. (2015) described the spatial regularity effect of real-world objects in VWM, whereas we conducted a further examination of the specific phase during which the effect emerged, and we tested the encoding specificity and perception-alike hypotheses by utilizing a sequential presentation condition. The core difference between the two hypotheses is whether the effect of spatial regularity occurs during the VWM maintenance period. Kaiser et al. (2015) used a simultaneous presentation condition, which could not exclude the possibility that the VWM maintenance phase might benefit from spatial regularity. In contrast, our study examined this possibility by using a sequential presentation condition in which participants could only acquire adaptive spatial regularity in the second memory array when they could manipulate VWM representations of the other half of the objects that had disappeared from the screen. Our results showed that the spatial regularity effect emerged only under the simultaneous presentation condition and not the sequential presentation condition, suggesting that the participants could not employ spatial regularity to integrate object pairs online. Consequently, these results provide strong support for the encoding specificity hypothesis.

We also consistently found that participants showed no significant difference in their sensitivity to with-regularity and without-regularity object pairs in the sequential presentation trials across the four experiments with the same memory load. The combined analysis of the results in these trials, based on a relatively large sample, confirmed the disappearance of the spatial regularity effect. The results verified the stability of the encoding specificity hypothesis in experiments with differences in the memory loads, verbal rehearsal, sensory afterimages, or eye movements, which seemed to contradict the results of Gao et al. (2016). However, the stimuli differed between the current study and their study, as Gao et al. (2016) used much simpler stimuli (e.g. oriented arrows) while we applied real-world objects (e.g. a teapot) that contained ampler information and more complex integration principles. Previous studies have found that the mechanisms underlying the integration principles for simple stimuli and real-world objects differ (Kaiser et al., 2019). Simple perceptual integration principles influence VWM in a bottom-up way, and they automatically attract attention (Bharti, Yadav, & Jaswal, 2020), whereas the integration principles for real-world objects necessitate top-down attention capacity (Gronau & Shachar, 2014).

Neuroimaging studies have also provided evidence supporting the idea that integration principles for simple and complex objects function differently in VWM. Luria and Vogel (2014) examined the common fate principle of simple colors using contralateral delayed activity (CDA), an ERP component sensitive to the number of objects maintained in the VWM (Luria, Balaban, Awh, & Vogel, 2016). In the four-to-two condition trials, four colored squares moved in separate directions before they met. The small one overlapped the large one, and the two pairwise combinations continuously moved together until they disappeared. They found no significant difference between the CDA amplitude for this condition in the maintenance phase and for two colored squares moving independently throughout the memory array, thereby indicating that the common fate principle benefited from the online integration of simple colored squares. However, a colored square is only a single-dimensional feature. When the memory material consists of multidimensional objects or features, online integration can be very difficult. Balaban and Luria (2016) also investigated the possibility of online integration of a colored square and a tilted bar, using a similar procedure to that used in Luria and Vogel (2014). They did not find the integration of the multi-dimensional features during the maintenance process because the CDA was higher for the four-to-two condition than for the two-object condition.

Moreover, notwithstanding the use of simple stimuli (Pac-Man), Zhang and Du (2022) noticed that the obviously perceptive similarity principle could enhance VWM performance, whereas the relatively complex proximity principle could not in sequential presentation tasks. Taken together, the findings indicate that the utilization of integration principles in a sequential presentation depends partly on the complexity of the stimuli and principles. In our study, the real-world objects were far more sophisticated than the colored squares and multi-feature conjunctions used in previous studies, and spatial regularity was also not as obvious as the similarity Gestalt principle. Therefore, we can propose the reasonable assumption that the participants were unable to integrate real-world objects easily during the maintenance process.

A natural question that arises is why the rate of failure of online integration might increase as the complexity of memory objects and integration principles escalate. The Gestalt principle essentially works automatically, whereas the use of spatial regularity demands comparisons between perceptual or VWM representations and prototypes in long-term memory. One possibility is that the VWM is actually unable to allocate enough resources to spatial regularity during the memory process, and the stimuli are actually integrated during the perception process. In other words, VWM limitations could have caused integration failures in both the maintenance phase and the working memory encoding phase, but the participants integrated the simultaneously presented object pairs once they saw them. Therefore, perceptual encoding provided pairs of integrated objects to the VWM system. However, our study did not test this hypothesis. Moreover, perceptual encoding and working memory encoding usually entwine tightly in VWM tasks and daily life; therefore, distinguishing them seems less urgent.

Furthermore, the inevitable increase in eye movements in sequential presentation trials might impede online spatial integration. Sequential presentation and vertical object configuration cause more passive eye movements (Burke, Allen, & Gonzalez, 2012), which could disrupt the configural process because spatial representations have to be updated with each movement to maintain perceptual stability (Martin et al., 2017). The results of experiment 4 indicated that eye movements had no positive impact on the integration of real-world object pairs; however, we could not exclude a negative impact, and the increased eye movements might have been the reason why the spatial regularity effect disappeared in the sequential presentation. For example, participants moved their eyes more during sequential presentation than they did during the encoding phase when all the information needs to be parsed at once. As a result, they may have more often foveated the top and bottom objects separately when they were presented one by one rather than fixating them as a pair when they were presented alongside. This may have cancelled out visual field-specific regularity effects. However, Gao et al. (2016) also used a sequential presentation design similar to the one we used in our experiment, and they observed the online integration of simple stimuli. Therefore, inevitable eye movements during VWM maintenance caused by sequential presentation settings may not hinder the stimulus-driven integration of simple items but will interfere with top-down online integration using the spatial regularity of real-world objects. This study has provided sufficient evidence regarding an individual's inability to perform online integration of real-world objects based on their spatial regularity; therefore, future studies should use eye-tracking techniques to further investigate the mechanisms underlying the failure of this online integration of real-world objects.

Our study has some limitations. We still could not directly observe the subprocesses of VWM that are influenced by the spatial regularity of real-world objects. Future studies should use event-related potentials (e.g. CDA components) to visualize the specific VWM process that is influenced by spatial regularity. In addition, because we only used static pictures of real-world objects, we were unable to examine the dynamic properties of moving objects within the VWM. Previous studies have investigated integration within VWM through dynamic demonstrations of the integration of separate objects, and CDA has been used to reflect this active process (Balaban & Luria, 2016; Luria & Vogel, 2014). However, most of the findings were still constrained to the domain of simple stimuli. Future studies should pay attention to the dynamic properties of VWM in relation to real-world objects.

In conclusion, we found no spatial regularity effect in sequential presentation trials, regardless of memory load, indicating that individuals do not perform the online integration of real-world object pairs. Our results support the encoding specificity hypothesis rather than the perception-alike hypothesis.

## Supplementary Material

Supplement 1
