# Supplementary material for: Encoding specificity instead of online integration of real-world spatial regularities for objects in working memory
Source: J Vis. 2022 Aug 30;22(9):8. doi: 10.1167/jov.22.9.8 (PMC9437652; doi:10.1167/jov.22.9.8)
Supplement: Supplement 1 [file jovi-22-9-8_s001.pdf]

# Supplementary Materials

## Encoding specificity instead of online integration of real-world spatial regularities for objects in working memory

Xinyang Liu[1,2+], Ruyi Liu[1+], Lijing Guo[1], Piia Astikainen[2], Chaoxiong Ye[1,2,3,4*]

[1] Institute of Brain and Psychological Sciences, Sichuan Normal University, Chengdu, China;
[2] Department of Psychology, University of Jyvaskyla, Jyväskylä, Finland.
[3] Faculty of Social Sciences, Tampere University, Tampere, Finland;
[4] Center for Machine Vision and Signal Analysis, University of Oulu, Oulu, Finland.

+ Xinyang Liu and Ruyi Liu contributed equally to this work and should be considered as co-first authors.

* Correspondence should be addressed to Chaoxiong Ye, Institute of Brain and Psychological Sciences, Sichuan Normal University, Chengdu, 610068, China. E-mail: cxye1988@163.com.

## Visual material selection

Before the formal experiments, we conducted a judgment task to select the most suitable visual materials.

Twelve different categories of paired real-world objects with spatial regularity in the vertical direction were downloaded from the Internet. Each object consisted of three alternative images, resulting in 72 images. We conducted a judgment task in which the participants were asked to determine the spatial relationships between the presented objects. The main purposes of this experiment were to test the effectiveness of prepared spatial regularities (i.e., whether the participants could detect them) and to select the two most effective pairs of each category of real-world objects (two upper objects and two lower ones for each category) for use as stimuli in the formal experiments. Accuracy and reaction time were recorded.

If the participants were able to correctly detect the spatial regularity of the paired objects, the accuracy of object pairs with spatial regularity (e.g., a beach umbrella on a beach chair) would reveal a ceiling effect, and even very high accuracy rates in the without-regularity (with the reversed spatial relationship, e.g., a beach chair on a beach umbrella) and no-regularity (e.g., a mirror on a beach chair) (see Figure S1) conditions would indicate that the participants could precisely reject abnormal spatial associations. Based on the accuracies and reaction times for each pair of objects, we chose the two most appropriate pairs of images from each category of objects as the formal experimental materials.

### Methods

*Participants*

In total, 10 healthy undergraduates were recruited (7 females, 24.4 ± 7.6 years old) for the judgment task.

*Stimuli and procedure*

All images were decolorized and matched for size using Photoshop 2020. Images were presented against a gray (140, 140, 140, RGB) background on a 21-inch LCD

monitor (refresh rate: 75 Hz). A single object subtended a visual angle of about 3°. The experiment was programmed using E-prime 2.0.

At the beginning of the experiment, a pair of real-world objects was displayed in the center of the screen. We manipulated the grouping of object pairs to present three levels of stimuli: with-regularity pairs (70 trials), without-regularity pairs (70 trials), and no-regularity pairs (70 trials). Participants were asked to identify the spatial relationships between the displayed objects and to press number "1" if the objects had spatial regularity, "2" if they had reversed spatial regularity, and number "3" if no spatial relationship was evident (see Figure S1). The total duration of the experiment was about 10 min.

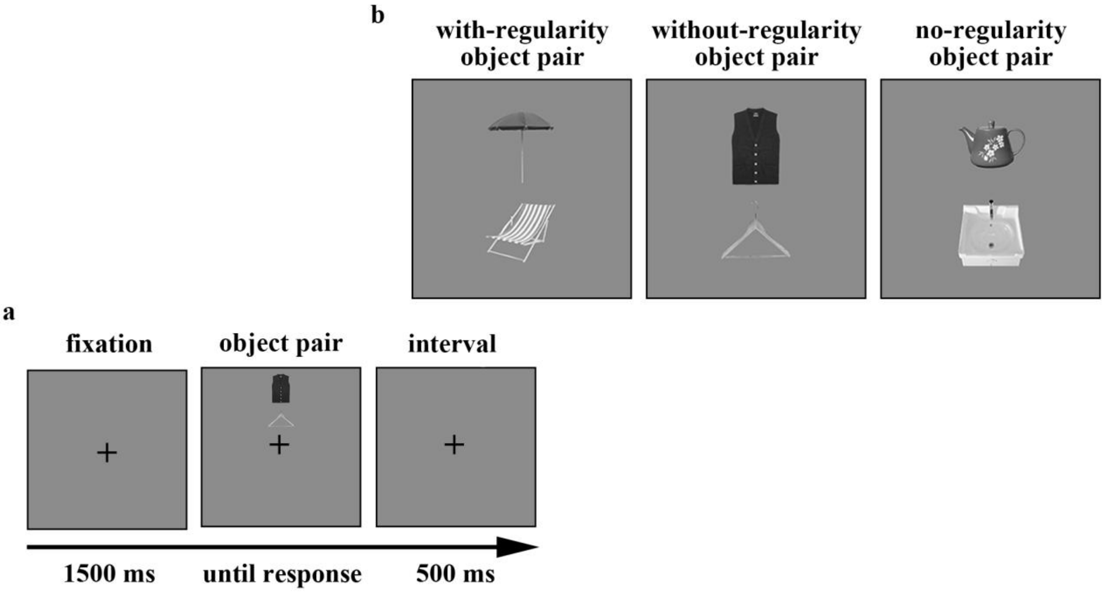


***Figure S1.*** (a) The main procedure for the judgment task and (b) the three conditions.

*Data analysis*

A one-way (pair configuration: with-regularity vs. without-regularity vs. no-regularity) repeated measures ANOVA was conducted for the accuracy.

## Results and discussion

The mean accuracy rates across the three conditions were all very high (with-regularity: $0.97 \pm 0.03$; without-regularity: $0.96 \pm 0.03$; no-regularity: $0.97 \pm 0.02$), showing a ceiling effect. The results of the one-way repeated measures ANOVA on

the accuracy data revealed no significant main effect of pair configuration, $F(2, 7) = 0.85$, $p = 0.44$, $\eta_p^2 = 0.09$. These results suggest that the participants were able to correctly detect spatial regularity between the presented stimuli. In addition, the accuracy rate was very high for each with-regularity object pair ($0.97 \pm 0.05$), which also showed a ceiling effect.

All of these results demonstrated that the participants could identify whether spatial regularity existed among real-world objects, regardless of the grouping pattern, and that the spatial regularity of our optional stimuli was obvious.

We selected the best experimental materials from among these optional stimuli by excluding a pair of images from each category of objects. To address the ceiling effect on accuracy, we used the reaction times in the trials with correct response to select the most effective with-regularity object pairs. We first chose the two pairs identified most quickly (shortest response time) among nine associations (3 upper objects $\times$ 3 lower objects) of each category. If we obtained four objects, the selection was considered complete, whereas if we obtained only three (i.e., lacking an upper or a lower object), we chose one of the fastest pairs from those containing the objects we had picked out, bringing the total to two upper objects and two lower ones. Using these criteria, we ultimately obtained 48 images (two pairs for each category of objects) as the material for the formal experiments.

## Relationship between visual working memory (VWM) capacity and the spatial regularity effect

In Experiment 2b, we examined whether individual differences in VWM capacity might influence the spatial regularity effect for sequentially presented stimuli by asking participants to engage in a color change detection task to test their VWM capacity after they had completed the main task.

### Methods

*Participants*

The same 25 participants (19 females, 19.76 ± 1.54 years old) from Experiment 2b were recruited.

*Stimuli and procedure*

Squares of different colors were presented against a gray (114, 114, 114, RGB) background on a 21-inch LCD monitor (refresh rate: 75 Hz). A single square subtended a visual angle of about 3°, and every two squares kept a distance of at least 1.5°. The experiment was programmed using E-prime 2.0.

At the beginning of the experiment, six squares of different colors were displayed randomly on the screen around the fixation for 200 ms, followed by an interval with a fixation for 1000 ms. The test array then began, and one colored square appeared in one of the six locations in the memory array. Participants were asked to identify whether the color in the test array was identical to the color of the same location in their memory and to press “J” if the color had changed; otherwise, they were to press “F.” The color in the test arrays of half the trials was identical to that of the memory array, whereas it was changed in the other half. The test array would not disappear unless the participant pressed a key or 2500 ms had passed (see Figure S2). This capacity experiment contained 100 trials, and the total duration was about 10 min.

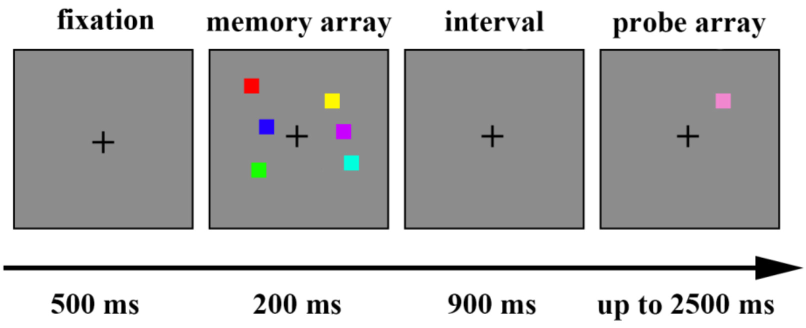


***Figure S2.*** The main procedure for VWM capacity measurement.

*Data analysis*

The VWM capacity ($K$) of each participant was quantified based on their results in the VWM capacity measurement test. The standard formula proposed by Cowan (2001) was applied: $K = N \times (H - F)$, where $K$ is the VWM capacity, $N$ is the size of the array (i.e., six in the present study), $H$ is the hit rate or proportion of correct responses when a change is present, and $F$ is the false alarm rate or proportion of incorrect responses when no change is present.

The spatial regularity effect for each participant was quantified by calculating the spatial regularity index (SRI) by subtracting the *d*-prime score for the without-regularity condition from the *d*-prime score for the with-regularity condition.

The two-tailed Pearson's $r$ correlation coefficients between $K$ and SRI were calculated under each memory load condition to investigate the relationship between VWM capacity and the spatial regularity effect.

## Results and discussion

The Pearson's $r$ correlations revealed no significant association between $K$ and SRI in the two-pair condition, $r = 0.134$, $p = 0.513$ or in the three-pair condition, $r = -0.108$,

*p* = 0.606. These results indicate that individual differences in VWM capacity had no influence on the degree of online integration, regardless of memory load.

## Reference

Cowan, N. (2001). The magical number 4 in short-term memory: A reconsideration of mental storage capacity. *Behavioral and Brain Sciences*, 24(1), 87–114.
